# A Technology-Aided Program to Support Basic Occupational Engagement and Mobility in Persons with Multiple Disabilities

**DOI:** 10.3389/fpubh.2017.00338

**Published:** 2017-12-11

**Authors:** Giulio E. Lancioni, Nirbhay N. Singh, Mark F. O’Reilly, Jeff Sigafoos, Gloria Alberti, Francesca Campodonico, Viviana Perilli, Valeria Chiariello, Carmen Zimbaro

**Affiliations:** ^1^University of Bari Aldo Moro, Bari, Italy; ^2^Medical College of Georgia, Augusta University, Augusta, GA, United States; ^3^University of Texas at Austin, Austin, TX, United States; ^4^Victoria University of Wellington, Wellington, New Zealand; ^5^Lega F. D’Oro Research Center, Osimo, Italy

**Keywords:** technology-aided program, occupational engagement, mobility, heart rates, auditory cues, visual cues, multiple disabilities

## Abstract

**Background:**

Persons with severe/profound intellectual and multiple disabilities tend to be passive and sedentary. Promoting their occupational engagement and mobility (i.e., indoor walking) can help to modify their condition and improve their environmental input, health, and social image.

**Aim:**

This study assessed whether a technology-aided program was suitable to (a) support independent occupation and mobility in eight participants with intellectual and sensory disabilities and (b) eventually increase the participants’ heart rates to levels considered beneficial for them.

**Method:**

The program, which involved a computer system regulating the presentation of auditory or visual cues and the delivery of preferred stimulation, was introduced according to a non-concurrent multiple baseline design across participants. The auditory or visual cues guided the participants to collect objects from different desks and to transport them to a final destination (i.e., depositing them into a carton). Preferred stimulation was available to the participants for collecting and for depositing the objects.

**Results:**

During the program, all participants had an increase in their independent responses of collecting objects and transporting them to the final destination. Their heart rates also increased to levels reflecting moderate-intensity physical exercise, potentially beneficial for their health.

**Conclusion:**

A program, such as that used in this study, can promote occupational engagement and mobility in persons with multiple disabilities.

## Introduction

A basic objective of rehabilitation and care centers for persons with multiple (e.g., intellectual and sensory) disabilities is to set up conditions suitable for promoting their occupational engagement and mobility (i.e., indoor walking) (1–5). Occupational engagement and mobility can be considered critical requisites to (a) counter the persons’ tendency to be passive, with possible benefits for their physical condition, (b) provide them a chance of meaningful actions, (c) increase their opportunities of positive environmental input, and (d) improve their social image (6–11).

To pursue successful occupational engagement and mobility with them one needs to set up intervention strategies suitable to deal with their typical weaknesses, that is, limited object manipulation and activity skills, minimal engagement motivation, and problems with spatial orientation and transition from one response/place to the next (12–14). An intervention approach directed at reducing the impact of the aforementioned weaknesses and enabling the persons to reach occupation and mobility independent of specific staff supervision (i.e., in a practically sustainable manner) would need to rely on technology-aided programs (12, 15–18).

One of the technology-aided programs recently assessed in this area (12) was designed to help the participants, (a) transit from one simple activity (e.g., assembling or sorting a few objects) to the next of a series through sound or light cues and (b) obtain positive stimulation in relation to each activity. Cues and stimulation were regulated automatically. A second technology-aided program (16) was designed to help the participants, (a) collect objects from different desks, which were indicated by automatically delivered sound or light cues, (b) transport those objects to a final destination (i.e., depositing them into a specific container), and (c) obtain automatically regulated stimulation for collecting and depositing the objects. This second program emphasizes mobility more than the first and is suitable for participants with no particular activity skills.

The evidence available regarding the aforementioned programs is quite encouraging. In fact, the participants learned to use the technology and reached independent occupation and mobility. Notwithstanding the positive evidence, the fact that only a few participants were involved in the two programs recommends caution in drawing conclusions and stresses the need for new research (19). The purpose of this study was to extend the assessment of the second program, in line with the notion of systematic replication (19). In practice, this study implemented the same type of program conditions, but using different spatial arrangements and longer (i.e., 15 min) sessions, thus providing extra occupation and mobility opportunities, with eight new participants. Data were to clarify whether those conditions and the new set up were suitable for promoting the participants’ independent occupation and mobility, and also led to relevant increase in their heart rates. Such increases (not investigated in the aforementioned studies) were viewed as a positive sign, with potentially beneficial implications for the participants’ health (20–22).

## Materials and Methods

### Participants

The eight participants, whose pseudonyms, ages, and sensory impairments are reported in Table 1, attended rehabilitation and care centers, and represented a convenience sample (23). They had congenital encephalopathy with intellectual disability and limited residual vision (Judy and Casey) or total blindness (all the others). Judy was also affected by severe hearing loss. Their intellectual disability was reported by the centers they attended to be within the severe or severe/profound range. Yet, no formal testing or IQ scores were available. Vineland age equivalences for their daily living skills were about or below 3 years (24). Besides the intellectual and sensory disabilities, the participants shared three other conditions critical for their inclusion in the study. First, they were able to walk without support and were known to have preferred environmental stimuli (i.e., stimuli that could serve as motivating events during the study). Second, while lacking speech abilities, they could use a few gestures and/or understand a few verbal instructions dealing with common objects and activities. Third, they could make simple use of objects (e.g., take objects from a table, transport, and put those objects into a container) when they were assisted *via* spatial orientation cues indicating the objects’ locations and received positive stimulation for their performance. Without assistance, they tended to be passive and sedentary. While the participants could not be interviewed about their willingness to enter the study, staff and legal representatives had expressed their interest for a technology-aided program that could promote the participants’ independent object use and mobility and increase their heart rates. The legal representatives had also provided written informed consent for the participants’ involvement in this study. The study complied with the 1964 Helsinki declaration and its later amendments and was approved by the Ethics Committee of the Lega F. D’Oro, Osimo, Italy.

**Table 1:** Participants’ pseudonyms, ages, and sensory impairments.

Participants	Ages (years)	Sensory Impairments
Adrian	51	Total blindness
Judy	48	Limited residual vision and severe hearing loss
Doug	27	Total blindness
Kevin	39	Total blindness
Holly	45	Total blindness
Randy	14	Total blindness
Ginny	31	Total blindness
Casey	24	Limited residual vision

### Setting

The study was carried out in the rehabilitation and care centers that the participants attended. Those centers were part of the same Italian organization devoted to the treatment of persons with multiple disabilities and shared the same professional values and daily arrangements.

### Sessions

The study included baseline, intervention, and rest sessions. Baseline and intervention sessions lasted 15 min or until any occupation and mobility response (see below) started before that time limit had been completed, and typically occurred one to three times a day, covering periods of up to 4 months. Rest sessions lasted 3–5 min, preceded the start of at least one of the daily intervention sessions, and served as the basis for determining the level of heart rate increase during the intervention sessions.

### Research Assistants

Five research assistants experienced in the use of technology-aided programs with persons with multiple disabilities were in charge of the sessions, and thus, set up the technology, provided verbal and physical prompting, and carried out data recording. To promote their procedural reliability, preliminary preparation meetings had been conducted in which their role was illustrated through the use of demonstration videos and direct role modeling.

### Data Recording

Data recording concerned the participants’ occupation and mobility responses (during the baseline and intervention sessions) and their heart rates (during the rest and intervention sessions). For accuracy purposes, any occupation and mobility response was divided into two partial sequences, which were recorded separately. One partial sequence consisted of the participant walking to a desk and taking an object there. The other partial sequence consisted of the participant transporting that object and depositing it into the carton of the last desk. A partial sequence was recorded as correct if it was performed accurately and independently. The heart-rate measure was recorded *via* a wristwatch with built-in heart-rate monitor (providing the mean heart-rate value for each session) or with a fingertip pulse oximeter (see below). Inter-rater reliability on recording the partial occupation and mobility response sequences was checked in about 20% of the participants’ sessions. Agreement (which required that the research assistant and reliability observer report the same number of correct partial sequences) was registered in more than 90% of the sessions for all participants.

### Stimulation

Positive stimulation occasions were scheduled during the intervention sessions, at the end of each of the two partial response sequences (i.e., as the participants reached a desk and collected an object and as they deposited the object in the carton of the last desk). The stimuli the participants received consisted of music and songs, which could be combined with praise statements, or vibration and lights. The stimuli had been selected following a stimulus preference screening, during which two or three 5- or 10-s segments of each music piece or song, light display, or vibration event available had been presented non-consecutively for at least 15 times. A stimulus was selected for use during the intervention if the research assistant and staff in charge of the screening agreed that it produced positive reactions (i.e., alerting or smiling) in over 60% of the presentations.

### Materials and Technology

The setting was fitted with four to six desks. Each desk except the last contained a series of familiar objects (e.g., bottles of water or cases with utensils). The last desk contained a carton in which the participants deposited the objects collected from the previous desks. The distance between the desks with familiar objects and the desk with the carton varied between 9 and 19 m. The technology at each of the desks included an electronic box with an optic sensor. The box served to provide verbal orientation cues and feedback (visual orientation cues for Judy), as well as preferred stimuli. The stimuli were delivered directly *via* the box (or *via* light and vibration devices connected to it for Judy). Boxes and optic sensors were linked to a remote control unit, which regulated their functioning. The technology also included the aforementioned wristwatch with built-in heart-rate monitor (i.e., Garmin Vivosmart HR or TomTom Runner Cardio) and the fingertip pulse oximeter (Oximeter OXY-6, Gima). The oximiter was used for only one participant (i.e., Casey) who had problems wearing the wristwatch. For this participant, two or three finger pulse readings were taken at each session and averaged. Prior to the study, each of the wristwatches was tested against a heart-rate monitoring device relying on a chest strap transmitter (Geonaute Onrhythm 310, Decathlon). A total of 87 test sessions were carried out for the Garmin device and 95 sessions for the TomTom device. The sessions lasted between 5 and 15 min and were carried out by three research assistants. The heart-rate values of the two wristwatch devices were within a 3-point margin from those of the device relying on the chest strap transmitter in more than 90% of the sessions, thus indicating that the wristwatches could be used dependably.

### Experimental Conditions and Data Analysis

The study was carried out according to a non-concurrent multiple baseline design across participants who received four to nine baseline sessions prior to the start of the intervention phase (25). The number of baseline sessions for the single participants was preset, but extra sessions would be used if the participants’ frequency of correct partial response sequences at the last session was above five and exceeded the frequency of previous sessions (this condition never applied). The intervention sessions served to determine the effects of the technology-aided program on the participants’ correct partial response sequences and heart rates (i.e., compared to the baseline and the rest sessions, respectively).

The baseline and intervention frequencies of correct partial response sequences as well as the rest and intervention heart rates were summarized/graphed as means per session over blocks of sessions, and analyzed *via* paired *t*-tests (26). These tests were used to compare the participants’ mean intervention and baseline response sequences, and their mean intervention and rest heart rates. The participants’ rest and intervention heart rates were also related to the participants’ estimated maximum heart-rate levels. Rates reaching 50–70% of their maximum levels were considered to reflect moderate-intensity physical exercise (27, 28). The maximum heart-rate levels were computed using the formula: “210 − [0.56 × individual’s age] − 15.5” (29–31).

#### Rest Sessions

Participants received 32–75 rest sessions, which were carried out during periods in which they sat quietly. They wore the Garmin Vivosmart HR or TomTom Runner Cardio watch except for Casey who was checked twice with the oximeter.

#### Baseline Sessions

At the start of each of the four to nine baseline sessions, the research assistant guided the participants through some of the desks (which contained objects as well as inactive boxes and optic sensors), and then directed them toward the first desk. During the sessions, the research assistant used verbal and physical prompting if the participant failed to walk for over 1 min, did not reach any desk within about 2 min, or failed to take an object from the desk. At the end of the sessions, the research assistant presented the participant with praise and possibly a few seconds of music or an edible item.

#### Intervention Sessions

Participants received 80–117 intervention sessions. A total of 32–75 of those sessions were preceded by a rest session. The intervention sessions differed from the baseline sessions in that the technology was functioning. Every intervention session started with the activation of the box on one of the desks containing objects, which emitted verbal or visual cues. The verbal cues consisted of one- or two-word utterances occurring at intervals of about 7 s. The visual cues consisted of stroboscopic light flashes occurring at intervals of about 1 s. The participant was to reach the desk and take one of the objects available there. Reaching the desk activated the optic sensor and caused the box to provide a one-word feedback and shortly thereafter deliver music plus verbal praise (or lights and vibration for Judy) for about 5–10 s. At the end of this stimulation, the box at the last desk containing the carton started to emit cues. The participant was to transport and place the object in the carton. Placing the object in the carton triggered the optic sensor available there and led the box to provide a 20-s stimulation (i.e., music or songs with or without praise for each participant except Judy who received light and vibratory input). At the end of the 20-s stimulation, the box at another desk started to emit cues and the process continued until the session time had elapsed. The intervention sessions were preceded by six to eight practice sessions, during which the research assistant applied the verbal and physical prompting required to ensure that the participants would use the technology and perform the partial response sequences accurately.

## Results

**Figure 1:**
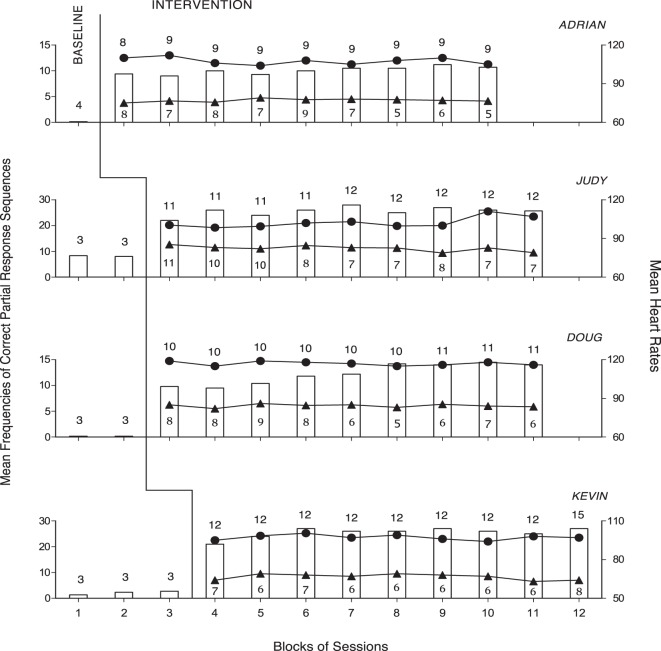
The four panels report the data for Adrian, Judy, Doug, and Kevin. The bars represent mean frequencies of correct partial response sequences per session over blocks of baseline and intervention sessions. The circles represent mean heart rates per session over the aforementioned blocks of intervention sessions. The number of sessions included in each block is indicated by the numeral above the bar. The triangles represent mean heart rates per session over blocks of rest sessions. The number of sessions included in these blocks is indicated by the numerals below the triangles.

**Figure 2:**
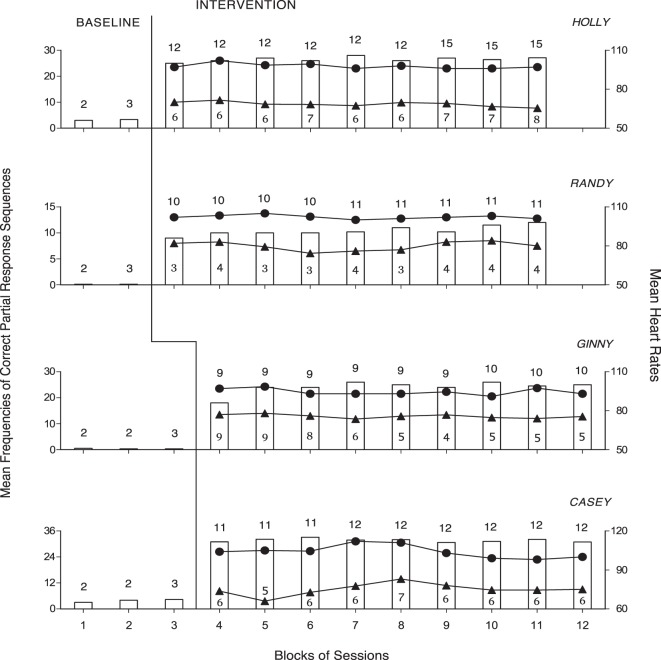
The four panels report the data for Holly, Randy, Ginny, and Casey. Data are plotted as in Figure 1.

Figure 1 summarizes the data for Adrian, Judy, Doug, and Kevin. Figure 2 summarizes the data for Holly, Randy, Ginny, and Casey. Bars, circles, and triangles represent correct partial response sequences, heart rates during intervention, and heart rates during rest, respectively. During the baseline, the participants’ mean frequencies of correct partial response sequences per session were below four except for Judy who had a mean frequency of about eight. During the intervention, those mean frequencies increased to about 10 (Adrian) and 32 (Casey) per session. The different frequencies reflected the participants’ differences in performance/ambulation speed and also different distances between desks. In fact, the participants were largely accurate and independent in their responding, and prompting from the research assistants was sporadic/negligible. A paired *t*-test indicated that the participants’ mean frequencies were significantly higher [*t*(7) = 8.41, *p* < 0.001] during the intervention than during the baseline (intervention, M = 20.76, SD = 7.89; baseline, M = 2.16, SD = 2.71).

The participants’ mean heart rates during the 80–117 intervention sessions varied between 94 (Ginny) and 116 (Doug). Their mean heart rates during the 32–75 rest sessions varied between 67 (Kevin) and 84 (Doug). A paired *t*-test indicated that the participants’ mean heart rates were significantly higher [*t*(7) = 15.68, *p* < 0.001] during the intervention than during the rest period (intervention, M = 102.67, SD = 6.37; rest period, M = 76.11, SD = 5.75). The participants’ mean heart rates during the intervention exceeded the 50% level of their maximum rates (i.e., indicating moderate-intensity physical exercise).

## Discussion

The results show that the program was helpful in fostering occupational engagement and mobility in persons with intellectual disability and blindness or limited residual vision and hearing impairment. These results extend preliminary evidence as to the possibility of providing extra opportunities of occupation and mobility to generally passive persons, with reduced staff time costs (2, 8, 12, 16, 32). The results also indicate that occupation and mobility can promote a valuable increase in the persons’ heart rates with possibly positive implications for their physical condition (17, 20, 33). In light of the above, a number of considerations may be in order.

First, the program seems to represent a fairly satisfactory answer to the question of how to ensure higher levels of occupation and mobility to persons with multiple disabilities without taxing demands on staff time (9, 34, 35). The program might also be considered relatively simple for staff to use and friendly toward the participants (34, 36–39). The cost of the technology employed in the study could be estimated at about 2,500 US dollars, but cheaper versions might be envisaged by relying on the use of smartphones linked to commercial sound boxes (40, 41).

Second, the participants’ successful performance across the intervention sessions may find two general explanations. Those explanations concern (a) the availability of reliable and consistent guidance to reach the destinations and transport the objects, which allowed the participants to avoid engagement and mobility failures (8), and (b) the presence of preferred stimulation, which was automatically delivered in a very regular manner contingent on the participants’ performance (42, 43).

Third, the study did not include any specific assessment of the participants’ level of satisfaction during the intervention sessions. Even so, positive elements are available with regard to this point. Indeed, the virtual absence of research assistants’ prompts during those sessions emphasizes the participants’ consistent level of self-determination and reflects their (a) ability to comfortably manage session requirements and (b) strong motivation to respond (i.e., most likely due to the preferred stimuli available during the sessions) (35, 37, 44). These elements of self-determination and motivation together with anecdotal reports of participants’ smiles during the sessions might be taken to (a) suggest a condition of control and possible satisfaction, (b) dispel any realistic hypothesis of performance strain and anxiety, and (c) reassure about the acceptability of the intervention conditions and related technology.

Fourth, the impact of seemingly benign (anxiety-free) intervention sessions on the participants’ heart rates can be viewed as encouraging and clinically relevant. This study might represent a general illustration of how a combination of simple occupational engagement and ambulation can serve to promote functional physical exercise. It is reasonable to assume that periods of simple occupational engagement and ambulation supported *via* assistive technology could be incorporated into the persons’ daily schedule without major practical problems. Repeating those periods so as to reach the suggested 30 min daily regimen of moderate physical exercise could change the persons’ condition and improve their health outlook (20, 28, 45, 46).

Fifth, one of the several limitations of this study is the relatively small number of participants involved. Obviously, new studies with additional participants are warranted to verify the suitability of the program and the reliability of the findings (19, 47). Another limitation is the lack of specific evidence about the (a) participants’ satisfaction (e.g., indices of happiness or levels of cortisol) during the sessions and (b) physical benefits of the increased activity and heart rates (17, 48). In an attempt to address the latter point, one should probably monitor dimensions, such as muscle tone, body fluids regulation, mood, and sleep behavior (17, 34, 49, 50). A third limitation is the lack of a social validation assessment aimed at determining the opinion of staff about the program’s impact and usability within daily contexts (51, 52). Such an opinion might significantly add to the data and partly predict the future adoption of the program in daily settings (37, 39). Another apparent limitation is the lack of reliability (procedural fidelity) checks on the research assistants’ performance. In fact, research assistants’ experience and preliminary preparation were thought likely to guarantee procedural fidelity. While this view may be acceptable, the use of reliability checks remains an important methodological requirement (53).

In conclusion, the program seemed effective in fostering occupational engagement and mobility in persons with intellectual and sensory disabilities. Notwithstanding the positive results, caution should be used in drawing conclusions given the limitations of the study. New research needs to address those limitations and gain wider evidence about the effectiveness of the program and its acceptability and usability within daily contexts. Research efforts may also be directed at upgrading the technology available so as to make it suitable for a larger variety of users and ensure that its cost is easily affordable for rehabilitation and care centers (54, 55).

## Ethics Statement

Appropriate institutional board approval and written informed consent were obtained for the study. All procedures performed were in accordance with the ethical standards of the institutional and/or national research committee and with the 1964 Helsinki declaration and its later amendments or comparable ethical standards.

## Author Contributions

GL, NS, MO, and JS were responsible for setting up the study, acquiring/analyzing the data, and writing/editing the manuscript. GA, FC, VP, VC, and CZ contributed in acquiring and analyzing the data and editing the manuscript.

## Conflict of Interest Statement

The authors declare that the research was conducted in the absence of any commercial or financial relationships that could be construed as a potential conflict of interest.
